# Intertemporal Decision‐Making in Health Behaviours: A Conceptual Analysis

**DOI:** 10.1155/jonm/9712444

**Published:** 2026-05-19

**Authors:** Sisi Fan, Xinyi Lai, Jun Ma, Yu Zhu, Xuelin Xiao, Yaqin Zhou, Honghong Wang

**Affiliations:** ^1^ Xiangya School of Nursing, Central South University, Changsha, China, csu.edu.cn

**Keywords:** concept analysis, delay discounting, health behaviours, health-related decision preference, intertemporal decision-making, time preference

## Abstract

**Aim:**

To report a conceptual definition of intertemporal decision‐making in health behaviours.

**Background:**

Decisions regarding health behaviours—such as whether to smoke or exercise—occur daily. As these decisions involve trade‐offs between short‐term and long‐term outcomes, conceptualising them within an intertemporal context is crucial for understanding the mechanisms underlying health behaviours and advancing related research.

**Methods:**

This study employed Walker and Avant’s (2019) concept analysis technique. A systematic search was conducted across databases including PubMed, EMBASE, MEDLINE, CINAHL, APA PsycINFO, EconLit with Full Text and Scopus from the date each database was available to March 2025. Additionally, dictionaries, grey literature and reference lists were reviewed to identify relevant literature.

**Results:**

A total of 49 papers were included. Intertemporal decision‐making in health behaviours refers to the process through which individuals make trade‐offs and choices between the short‐term and long‐term outcomes of health‐related behaviours, with the consequences encompassing both different aspects (health aspect and nonhealth aspect) and different signs (costs and benefits). Throughout this process, individuals’ dynamic preferences in health‐related decisions ultimately determine their behavioural choices.

**Conclusion:**

This study developed a universal definition and conceptual model for ‘intertemporal decision‐making in health behaviours’, which aids in comprehensively interpreting individuals’ health behaviours. This conceptual analysis lays the foundation for future research, education, practice and policy, which also contributes to improving healthcare practices and global health.

**Implications for the Nursing Management:**

We recommend that nursing managers guide nursing teams to integrate patients’ health‐related decision preferences into care assessments, which promotes evidence‐based precision care practices and optimises medical resource allocation.

## 1. Introduction

The significance of health behaviours is self‐evident, yet substantial challenges persist in implementing such behaviours. According to reports from the World Health Organization, over 1.25 billion people worldwide currently use tobacco [1], nearly one‐third (31%) of adults globally fail to meet recommended physical activity levels [2] and approximately 3 billion people engage in unhealthy dietary practices [3]. Unhealthy behaviours significantly elevate risks of cardiovascular diseases, cancers and premature mortality, imposing substantial medical and economic burdens [4–6]. Understanding why people make irrational decisions in their health‐related behaviours is key to solving this problem. From a health economics perspective, decisions regarding health‐related behaviours inherently constitute intertemporal decision‐making as they involve trade‐offs and choices between long‐term and short‐term outcomes [7, 8]. For instance, while exercise demonstrably reduces long‐term risks of obesity, cardiovascular diseases and cancer, it requires an immediate investment of time, energy and effort [9, 10]. Individuals who assign greater weight to long‐term benefits are more likely to engage in exercise, whereas those prioritising short‐term costs tend to avoid it [11, 12]. Such intertemporal decision‐making in health behaviours occurs daily, such as whether to smoke, get vaccinated or drink. Analysing these decision‐making mechanisms can elucidate behavioural drivers and inform targeted interventions, thereby offering actionable insights for improving public health outcomes.

## 2. Background

Intertemporal decision‐making, also known as intertemporal choice, originates in economics. It refers to individual’s trade‐offs and choices between the costs and benefits occurring at different time points, especially those in the present and future [13]. For instance, when making an investment decision, one needs to make trade‐offs between current cost and future returns. Similarly, decisions regarding health behaviours involve trade‐offs between present costs (money, energy and time) and future health benefits (lower morbidity and mortality) [14]. Current research on intertemporal decision‐making in health behaviours has yielded preliminary results. Studies have identified clear decision preferences among individuals when making intertemporal decisions about health behaviours, with these preferences primarily stemming from the influence of time frames [15–17]. From a health economics perspective, people devalue future health outcomes due to their delayed realisation—a phenomenon termed delay discounting [18]. Individuals with higher discount rates demonstrate more severe depreciation of future health consequences and are more likely to adopt health risk behaviours [19]. For example, in obesogenic environment, individuals with higher discount rates more severely underestimate the value of improved future BMI and are thus more likely to consume unhealthy food [20]. From a psychological perspective, people exhibit different preferences for short‐term versus long‐term outcomes; individuals who prefer short‐term outcomes often encounter self‐management challenges [14, 21, 22]. For instance, among individuals newly diagnosed with type 2 diabetes, those who prefer short‐term comfort are generally less inclined to adopt healthy diet [23]. These findings demonstrate the well‐supported intertemporal nature of health behaviour decisions. Therefore, a clear and comprehensive understanding of the connotation of intertemporal decision‐making in health behaviours is necessary.

It is worth noting that although studies on intertemporal decision‐making in the health field have proliferated since the 21st century, controversies have persisted. Firstly, the domain characteristics of healthy behaviours have not been fully addressed. Health is a unique commodity traded differently from money. Specifically, researchers find that individuals discount health more severely than money [9, 24]. Importantly, certain peculiarities in health behaviours cannot be explained by economic intertemporal decision‐making models. A case in point is the observed violations of stationarity in health‐related intertemporal decision‐making, challenging the quasi‐hyperbolic discounting model from economics [14, 25]. These findings indicate that economic intertemporal choices inaccurately capture the mechanisms of health‐related intertemporal choices. Intertemporal decision‐making in health behaviour operates through distinct mechanisms. Clarifying these mechanisms is essential for accurately understanding health behaviour and designing targeted interventions. Secondly, academic consensus remains elusive regarding theoretical frameworks, methodologies, tools and results. The options‐based approach employs delay discount rates to quantify decision‐making preferences in health behaviours, utilising standardised measures such as sexual behaviour and substance‐related discount tasks [17, 26, 27]. Conversely, the attribute‐oriented paradigm reflects preferences through time perspective, using validated instruments including the Consideration of Future Consequences (CFC) Scale for dietary and physical activity contexts [7, 8, 28]. Regarding research conclusion, some research indicates that the discount rate for health losses exceeds that for health gains [26, 29], whereas others propose the inverse [24, 30]. These inconsistencies undermine research validity and constrain practical applications. Clarifying the concept of intertemporal decision‐making in health behaviours is crucial for scientifically defining the theoretical framework and developing measurement paradigm, thereby generating reliable evidence and advancing the field.

Overall, the significance of intertemporal decision‐making in health behaviours has been widely recognised, yet its comprehensive conceptualisation within the realm of healthcare remains unexplored. When a conceptual practice lacks clarity, it can create confusion about how to implement it in the clinic and how to integrate it into research, policy and education [31]. Therefore, clarifying the concept of ‘intertemporal decision‐making in health behaviours’ is of milestone significance for understanding the mechanisms of health behaviours and guiding the follow‐up research and practice. The main objective of this study is to clarify the concept to comprehend how individuals make decisions regarding health behaviours, thereby informing improvements in clinical nursing practice. A secondary goal is to explore the differences between intertemporal decision‐making within the healthcare domain and that within the economic domain to help guide research on this topic in healthcare.

## 3. Methods

### 3.1. Concept Analysis Approach

This concept analysis was conducted using Walker and Avant’s methodology to inductively identify the antecedents, attributes and consequences of intertemporal decision‐making in health behaviours. Furthermore, this approach was employed to determine the conceptual applications within existing literature and describe established measurement tools, thereby establishing a theoretical foundation for future research and practice. Walker and Avant’s [31] method applies a recognised methodological framework and involves (1) selecting a concept; (2) determining the aims of the analysis; (3) identifying all uses of the concept; (4) determining its defining attributes; (5) identifying a model case; (6) identifying additional cases; (7) identifying antecedents and consequences and (8) identifying empirical referents [31].

### 3.2. Sample Selection and Data Collection Procedures

#### 3.2.1. Data Sources and Search Strategy

After consultation with the librarian, a comprehensive search strategy involving databases, dictionaries, grey literature and reference lists was developed to gather relevant literature for this analysis. Firstly, we consulted prominent dictionaries such as the Oxford English Dictionary, Merriam‐Webster Dictionary and Collins English Dictionary to identify possible uses and definitions of ‘intertemporal decision‐making’ and ‘health behaviour’. Next, we searched across international online databases including PubMed, EMBASE, MEDLINE, CINAHL, APA PsycINFO, EconLit with Full Text and Scopus from the date each was available to March 2025. These databases were chosen to ensure the accessibility of nursing and nonnursing literature because a wide range of intertemporal decision‐making has been documented in nonnursing literature. We used a combination of Medical Subject Headings (MeSH) terms (Delay Discounting and Health Behaviour), Boolean operators and key terms (Intertemporal Decision Making, Intertemporal Choice, Temporal Discounting, Time Preference∗, Health Related Behavio∗, Health Risk Behavio∗, Smoking, Eating, etc.). The retrieval is carried out using one library one strategy (Supporting Information 1). Additionally, we utilised reference lists from retrieved articles to identify potentially relevant studies that might have been overlooked in the database searches. Finally, grey literature was conducted through platforms such as Google, Google Scholar, the E‐Theses Online Service (UK), Open Grey, Grey Literature Report and ProQuest Dissertations and Theses (US and Canada) to access any theses, books and documents that are not published by commercial publishers.

#### 3.2.2. Inclusion and Exclusion Criteria

Eligibility criteria included citations that (1) were peer‐reviewed journal articles or practice guidelines; (2) mainly discussed or investigated intertemporal decision‐making in health behaviour and (3) were available in English. Citations were excluded if they (1) were unavailable articles (e.g. poster or conference abstract, not yet published or have been retracted); (2) involved animal subjects (e.g., rats); (3) primarily analysed neural mechanisms; (4) primarily analysed mathematical models and (5) substituted health‐related discounting with money‐related discounting.

#### 3.2.3. Data Collection and Analysis

**FIGURE 1:**
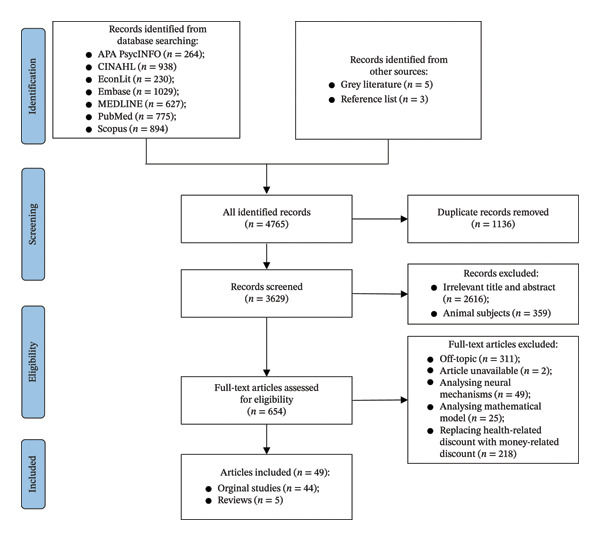
PRISMA flow diagram.

Fifty records were randomly selected for prescreening to determine the criteria for article selection. Subsequently, two researchers independently screened the literature according to the inclusion and exclusion criteria. An article was included only when both researchers considered meeting the criteria. Any disagreements between the two reviewers were reevaluated and resolved through discussions led by a third researcher until consensus was reached. The literature selection process was recorded using EndNote 20. Figure 1 shows the PRISMA flowchart of this process. This study searched 4757 studies through the database and obtained 3 records from the citation lists and 5 grey records from related websites, ultimately yielding a total of 4765 records. After deleting 1136 duplicate studies, excluding 359 animal studies and removing 2616 studies based on their titles and abstracts, a total of 654 studies were screened for eligibility. Among these, exclusions comprised the following: 311 studies off‐topic; 49 studies analysing neural mechanisms; 25 studies prioritising mathematical models over decision‐making dynamics; 218 studies substituting health‐related discounting with monetary metrics and 2 studies with unavailable full texts. The final synthesis comprised 49 publications, with 44 original studies and 5 reviews.

## 4. Results

### 4.1. Characteristics of Included Studies

**FIGURE 2:**
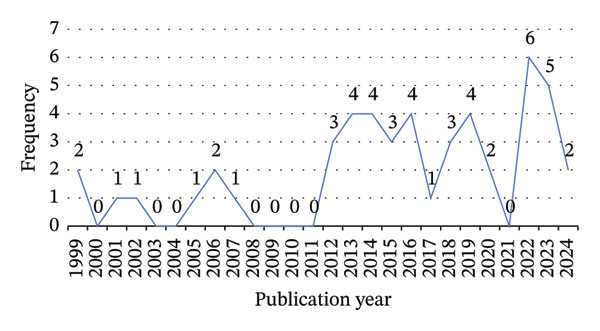
Frequency of publication year of included manuscripts.

The search yielded a total of 49 selected studies relating to the concept of intertemporal decision‐making in health behaviours. All articles were published between 1999 and 2024 in the United States (*n* = 24, 49.98%), United Kingdom (*n* = 7, 14.29%), Netherlands (*n* = 3, 6.12%), Norway (*n* = 1, 2.04%), Mexico (*n* = 1, 2.04%), Australia (*n* = 2, 4.08%), Germany (*n* = 2, 4.08%), France (*n* = 1, 2.04%), China (*n* = 2, 4.08%), Canada (*n* = 2, 4.08%), Denmark (*n* = 1, 2.04%), Iran (*n* = 1, 2.04%), Israel (*n* = 1, 2.04%) and Italy (*n* = 1, 2.04%). The study designs were mainly investigative studies. Three were experimental studies, and five were reviews. Figure 2 plots the number of studies published per year that were included in our analysis. There is a marked increase in the number per year from 2012, and 84% of all studies were published between 2012 and 2024. These 49 articles were mainly published in three journals: Experimental and Clinical Psychopharmacology (6; 12.25%), Appetite (5; 10.21%) and Journal of Behavioral Medicine (4; 8.20%). All these papers revolved around three journal clusters: nutrition, behavioural medicine and health psychology, with a pronounced tendency for co‐citation among them.

### 4.2. Uses of the Concept

‘Intertemporal’ is defined as ‘involving different time periods’ [32]. ‘Decision‐making’ denotes the act or process of making decisions [33]. ‘Health’ is defined as ‘beneficial to one’s physical, mental or emotional state’ and ‘conducive to or associated with good health or reduced risk of disease’ [34]. ‘Behaviour’ signifies the way in which someone conducts oneself or behaves [34]. The literature presents limited definitions of intertemporal decision‐making in health behaviours. Some studies describe the concept as the ‘trade‐off and choice between short‐term costs and long‐term health benefits’ [14, 29, 30]. This definition underscores a crucial distinction: unlike economic intertemporal decision‐making, the outcomes in health decisions are primarily health‐related rather than economic [14, 25]. However, this definition is confined to health‐protective behaviours and cannot explain intertemporal decisions in health‐risk behaviours since these behaviours typically involve long‐term health costs paired with short‐term nonhealth gains. This limitation reduces its applicability in health‐risk behaviour research. Consequently, most research on health‐risk behaviour can only focus on temporal frameworks of health decision. Scholars have proposed alternative constructs such as ‘health‐related delay discounting’, ‘time preference for health’, ‘health‐specific time perspective’ and ‘time orientation on health’ to reflect individuals’ decision preferences in health behaviours [14, 35, 36]. While these concepts capture core features of health behaviour decisions, they inadequately explain decision mechanisms by omitting crucial factors like the value and categories of health outcomes. This defect limits the depth of existing research and constrains it to superficial exploration of preference‐behaviour correlations while neglecting the underlying mechanisms of health‐related intertemporal decision‐making. In conclusion, these existing definitions fail to accurately and comprehensively reflect the complex connotations of intertemporal decision‐making in health behaviours. A closer examination of the definition is necessary to ensure its applicability and scientificity across various studies on health behaviours.

### 4.3. Antecedents

#### 4.3.1. Health‐Related Behaviours

Intertemporal decision‐making regarding health behaviours—such as whether to exercise, whether to smoke, when to go to bed or whether to measure blood pressure—occurs daily [37]. Health‐related behaviours are preconditions for decision‐making, as they require making choices among different behavioural patterns. Health‐related behaviours encompass patterns, actions and habits that contribute to health maintenance, restoration and improvement [38]. They include activities like alcohol use, tobacco use, physical activity, diet, sexual behaviour and self‐management behaviour [7, 9, 17, 27, 39, 40]. The options within these behaviours are often not singular. For example, in terms of tobacco use behaviour, individuals may choose to smoke or abstain from smoking. This is also the reason why people need to make decisions when faced with specific health‐related behaviours.

#### 4.3.2. Disproportionate Behavioural Outcomes

The outcomes of health‐related behaviours are multifaceted, encompassing different time points, aspects and symbols. Some health‐related behaviours incur immediate costs such as financial expenses, time, effort and foregoing pleasures, while the benefits—such as reduced morbidity and mortality—typically manifest over time [9, 15, 22, 40, 41]. For example, engaging in physical activity demands immediate costs like physical effort and time but offers delayed benefits in the form of improved health. Conversely, other related behaviours provide immediate benefits such as satisfaction and enjoyment, while incurring health costs like increased morbidity and mortality that are paid in a delayed manner [15, 19, 26, 27, 42]. For instance, unhealthy eating offers immediate benefits such as taste and satisfaction while the long‐term outcomes may include overweight or obesity. These disparities in outcomes introduce conflicts in decision‐making processes, which require people to make more complex trade‐offs when it comes to certain health behaviour.

#### 4.3.3. Individual Characteristics

The decision‐making style of individuals is closely linked to their personal characteristics. Men are generally more impatient than women, often favouring immediate outcomes [22, 43–45]. Decision makers who face greater economic pressure [15, 46, 47], belonging to lower social class [39], experiencing poorer accessibility to resources [16, 45, 48] or possessing lower cognitive function [49], tend to be more ‘short‐sighted’ and prefer behaviours that are beneficial in the present. Conversely, decision makers with higher education levels tend to be more ‘far‐sighted’ and are more inclined to adapt behaviours that are beneficial for the future [46, 47, 50]. For instance, in terms of dietary habits, women generally have a higher probability of adopting healthy eating behaviours than men. Conversely, individuals with higher economic stress, lower education and diminished cognitive show stronger tendencies towards unhealthy eating behaviours. In addition, factors such as age [22, 39], health status [20, 26, 27, 51], behavioural motivation [9, 52], desire [53], environmental stability [16] and risk perception [19, 53, 54] also contribute to decision‐making capacity and outcomes via their knock‐on effects on motivation.

### 4.4. Attributes

Analysis of the studies allowed us to identify key defining attributes. Table 1 reflects a complete and concise list of these attributes. Consequently, the defining attributes are outlined as follows:

1. Trade‐off between short‐term outcomes and long‐term outcomes
2. Trade‐off between other outcomes and health outcomes
3. Trade‐off between costs and benefits
4. Health‐related decision preferences drive the direction of choices
5. Health‐related decision preferences are inconstant

**TABLE 1:** Defining attributes.

Defining attributes	Sources
Trade‐off between short‐term outcomes and long‐term outcomes	[7–9, 20, 39, 49]
Trade‐off between other outcomes and health outcomes	[14, 19, 21–23, 26, 27, 44, 45, 51–53]
Trade‐off between costs and benefits	[9, 16, 25, 26, 29, 42, 50–53]
Health‐related decision preferences drive the direction of choices	
• Delay discounting	[24, 40, 43, 54–58]
• Time perspective	[16, 21, 51, 59]
• Time orientation	[7, 8, 15, 35, 46, 57, 60]
Health‐related decision preferences are inconstant	
• Sign effect	[24–26, 29, 42, 50, 53]
• Sequence effect	[20, 45, 50, 52]
• Magnitude effect	[20, 24, 41, 42]
• Domain effect	[24, 27, 45, 52, 61]
• Object of decision	[46, 62, 63]

#### 4.4.1. Trade‐Off Between Short‐Term Outcomes and Long‐Term Outcomes

Health‐related behaviours seem to pose a conflict between short‐term and long‐term outcomes of one’s actions, where health outcomes typically unfold gradually while other outcomes emerged much more promptly [9, 28, 41]. An important difference between people considering near and future outcomes is their level of mental representation: the near future is more concrete, whereas the distant future is more abstract. When health‐related choices are in the present and the immediate outcomes are concrete, they weigh more heavily than the abstract long‐term outcomes [37]. Those who are more informed about short‐term outcomes are more likely to engage in impulsive eating and unhealthy eating [20, 35, 39, 49], while those who can visualise long‐term outcomes are more inclined to engage in exercise and physical activities [7–9].

#### 4.4.2. Trade‐Off Between Other Outcomes and Health Outcomes

In any health‐related behaviour, health remains the primary outcomes, yet additional outcomes inevitably accompany these behaviours. Risky sexual behaviours involve significant health outcomes such as sexually transmitted infections and unwanted pregnancy, while they also provide satisfaction and enjoyment [19, 27, 44, 45]. Similarly, diabetes self‐management behaviours can prevent diabetic complications like kidney failure, stroke and heart attack but also come with self‐management effort, inconvenience and discomfort [14, 21–23]. Unlike financial intertemporal decision‐making, intertemporal decision‐making in health behaviours is no longer a trade‐off around money but revolves around other outcomes and health outcomes. Individuals who prioritise health tend to make ‘rational’ choices, opting for behaviours that promote health [51, 53]. Individuals who prioritise other outcomes are more ‘impulsive’ and are more likely to choose behaviours that damage their health [26, 27, 52].

#### 4.4.3. Trade‐Off Between Costs and Benefits

When an action brings all benefits without costs, its occurrence is typically unquestioned. However, health behaviours always involve both costs and benefits. Whether individuals engage in a health behaviour depends on their attitudes towards costs and benefits involved [16, 52]. For example, in the chronic pain population, opioid abuse—such as running out of prescriptions early, forging prescriptions, using other people’s opioids, purchasing illicit opioids or using substances (e.g., alcohol and cannabis)—is a common phenomenon [52]. Patients with chronic pain are more sensitive to loss, leading them to circumvent pain through painkiller use rather than minimising opioid intake to gain long‐term benefits (i.e., avoiding constipation) [26]. Conversely, individuals who enjoy exercise tend to value the benefits more, which makes them tolerate associated costs (time and discomfort) in order to obtain more health benefits (healthier BMI and better health status) [9, 51].

#### 4.4.4. Health‐Related Decision Preferences Drive the Direction of Choices

Health‐related decision preferences reflect individuals’ preferences for health outcomes in the temporal dimension and ultimately drive decision‐making direction [14, 22, 39, 56, 62]. Some studies operationalise these preferences through quantifying how individuals devalue delayed health benefits [14, 54, 58]. People with higher health discounts devalue future health more severely and are more likely to engage in health‐risk behaviours [40, 45, 52, 64]. Other studies reflect health‐related decision preferences through temporal constructs such as time perspective and time orientation. Time perspective for health represents cognitive and affective biases towards past, present and future health [16, 59]. Present perspective often leads to unhealthy behaviour [15], while a future perspective seems to motivate individuals to adopt healthier behaviours [51]. Time orientation reflects how individuals consider the distant outcomes of their health behaviours and their influence [7, 57, 60]. Those who prioritise future health outcomes are more likely to choose health protective behaviours [8, 35]. Overall, health‐related decision preferences drive the direction of choices in two ways. Those who prefer small, immediate health outcomes are more impulsive in decision and are more likely to engage in unhealthy behaviours. In contrast, those who prefer large, delayed health improvements tend to be more rational in decision and therefore more inclined towards healthy behaviours [25].

#### 4.4.5. Health‐Related Decision Preferences Are Inconstant

Due to the complexity of health, health‐related decision preferences are usually inconstant [22]. First, the degree to which people devalue future health outcomes depends on whether it is gains or losses, known as the sign effect [26]. Decisions regarding health‐risk and health‐protective behaviours cannot be generalised due to difference in delay discounting between health gains and losses [25, 29, 42]. Second, the sequence effect is prevalent in health contexts. Research indicates a tendency for increasingly steep discounting of delayed reward over time, which implies that health outcomes with longer waiting times are perceived as less valuable [20, 45, 50, 52]. Third, the magnitude effect demonstrates that delay discounting of health systematically decreases as magnitude of health gain increases [42]. Greater future health benefits from a behaviour result in reduced discounting of its delayed value [20, 24, 41]. Fourth, the domain effect encompasses diverse health behaviours. Health‐related decision preferences vary significantly across activities such as drinking, eating, disease treatment, smoking, health improvement and life‐saving [24, 27, 45, 52, 61]. Fifth, the identity effect warrants attention. Research indicates that individuals’ time preference for others’ health is higher than that for their own health, which means people are more cautious when it comes to their own health [62]. Consequently, preference shifts and preference reversal occur frequently in health‐related decision‐making [14].

### 4.5. Consequences

#### 4.5.1. Explicit Behaviour Pattern

The intertemporal decision‐making in health behaviour directly affects the performance pattern of health behaviour. Generally, each health behaviour contains two different behavioural patterns. For example, eating behaviour includes a healthy diet and an unhealthy diet [7, 20, 49]. Researchers categorise health‐related behaviours into health‐protective behaviour patterns and health‐risky behaviour patterns according to whether they are beneficial or harmful to health [10]. Individuals who value future health outcomes more than short‐term other outcomes during intertemporal decision‐making often engage in health‐protective behaviour patterns such as healthy eating, weight loss, exercise, smoking cessation and vaccination [8, 41, 56, 59, 65]. In contrast, individuals who value future health outcomes less than short‐term other outcomes tend to engage in health‐risky behaviour patterns such as condomless sex, promiscuity, drinking alcohol, unhealthy eating, opioid abuse, drug use and tobacco use [17, 44, 49, 50, 66].

#### 4.5.2. Health‐Related Outcomes

Many kinds of intertemporal decision‐making in health behaviours we made in the past and are making now collectively shape our future health outcomes. To be specific, health protective behaviours can produce positive health outcomes such as reduced disease incidence [22, 47], improved health status [25, 50, 51] and enhanced quality of life [58]. However, health‐risk behaviours often result in negative health outcomes such as accelerating disease progression [57], resulting in complication of diseases [9, 21], addiction disorders [17, 55], overweight and obesity [8, 49] and mental health issues [60].

### 4.6. Case

#### 4.6.1. A Model Case

Eric is an advertising designer in a big city, and his life here is colourful. A year ago, he was diagnosed as HIV antibody‐positive. Although he had no symptoms at the time, doctors still recommended that he start antiretroviral therapy as soon as possible. He learnt from his doctor that early treatment can help suppress the virus, reduce future complications and prolong life. However, antiviral treatment requires long‐term persistence and may be accompanied by some side effects (hair loss, skin darkening, obesity, etc.). Eric carefully evaluated the immediate costs and future benefits of starting antiviral therapy. He believes that future health is more valuable than current losses. After careful consideration, he decided to start treatment. However, 2 weeks later, he found that he began to lose hair and his skin turned dark. The visible changes in his appearance and the challenges of managing his condition publicly prompted Eric to reassess his priorities. Despite understanding the long‐term benefits of treatment, the immediate sacrifices seemed increasingly burdensome. He reassessed the immediate costs and future benefits of antiviral treatment. Compared with what he had to pay now, health in a few years seemed less attractive. Ultimately, Eric decided to stop antiretroviral therapy. In this case, whether to initiate the treatment entirely depends on the individual’s trade‐off between short‐term other costs and long‐term health benefits. This behaviour stems from personal preferences, which can shift as the perceived balance between costs and benefits changes. It illustrates how such a conceptual model typically functions in real‐world situations.

#### 4.6.2. Borderline Case

Zhang, a young white‐collar worker who commutes daily by car, has often neglected the habit of wearing a seat belt. He justified this by considering himself a skilled driver and found wearing a seat belt bothersome and uncomfortable. However, recently, he witnessed a traffic accident where the driver died because they were not wearing a seat belt. This incident made him realise the potential consequences of not wearing a seat belt, even though the likelihood of an accident was uncertain. Zhang is not someone who is adventurous; strictly speaking, he fears all forms of uncertainty. Consequently, he reassessed his driving habits and made a commitment to always wear a seatbelt while driving. In this scenario, the effectiveness of using seat belts presents inherent uncertainties. Zhang’s decision was based mainly on his perception of risk—a fear of uncertainty and a desire to avoid danger—not on careful weighing of definite outcomes. While such decisions fall under the broad category of ‘decision‐making’, they align more closely with risk‐based decisions, which differ from those involving trade‐offs between different time points.

#### 4.6.3. Contrary Case

Ben, a 56‐year‐old successful businessman, has been smoking for over 20 years. After a physical exam, his wife asked him to quit smoking. Ben protested vehemently but finally agreed to quit smoking, since his wife became upset. Despite his personal beliefs about quitting smoking, Ben acknowledges his deep love for his wife and respects her opinions unquestionably. He humorously tells his friends, ‘I love my wife dearly. Whether I quit smoking depends on whether she’ll be upset. After all, she’s always right, and I must do as she says’. In this case, Ben’s decision to quit smoking stems not from weighing and choosing between short‐term costs and long‐term health benefits but from external relationships and emotions. By allowing outside influence to override his autonomy, this case contradicts the core concept.

### 4.7. Empirical Referents

Empirical references provide clear, observable assessment metrics for health‐related decision preferences. At first, delay discounting task for health requires individuals to choose between a series of immediate, smaller health gains/losses versus delayed, larger health gains/losses [19, 20, 26, 42, 50]. For example, the sexual discounting task developed by Johnson and Bruner requires people to choose between immediate condomless sex and delayed condom sex, with delays presented in ascending intervals: 1 h, 3 h, 6 h, 1 day, 1 week, 1 month and 3 months [43]. Similarly, in smoking discounting task, individuals choose between longer survival time (*t* = 10 years) if abstaining from smoking and shorter survival time (*x*) while smoking, with *x* varying periodically [58]. These tasks enable researchers to compute discount rates, illuminating individuals’ tendencies towards immediate versus delayed gratification. However, these tasks are constrained in scope and vary significantly in parameter settings, thereby limiting the referability of their findings. Secondly, the CFC Scale, comprising CFC‐future and CFC‐immediate subscales, determined the importance an individual attaches to immediate results versus future results [67]. Based on that, the revised CFC‐Food and CFC‐Exercise are more in line with health scenarios [8, 35]. Moreover, the Zimbardo Time Perspective Inventory (ZTPI), a widely used tool with subscales like past‐positive, past‐negative, present‐fatalistic, present‐hedonistic and future orientation, has been applied to assess time‐related attitudes in health decision‐making [68]. While initially devised for broader contexts, the ZTPI has found application in studying intertemporal decision‐making in health behaviours [16, 21, 51, 59].

In this conceptual model, health‐related decision preferences are understood as an expression of an individual’s intertemporal preferences regarding health values, integrating both the assessment of health value and time orientation. From a content validity perspective, however, existing time‐oriented measures such as the ZTPI and CFC fail to adequately incorporate dimensions of health value assessment. Meanwhile, discounting tasks are limited by their experimental paradigm: the health values presupposed in such tasks often deviate from real‐world conditions, thus undermining their ability to capture the full meaning of health values. In contrast, instruments such as CFC‐Food and CFC‐Exercise explicitly integrate health value assessment with time orientation, demonstrating stronger content validity. Although their applicability across diverse health behaviours remains to be fully established, they serve as valuable prototypes for the further development of conceptually grounded measurement tools.

### 4.8. Conceptualisation

**FIGURE 3:**
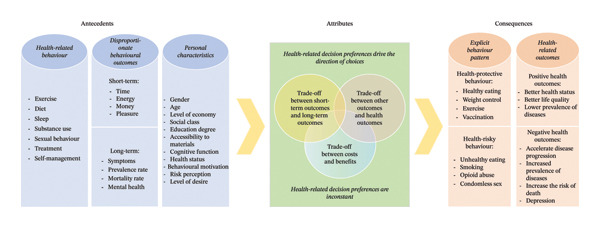
The conceptual model.

Based on our analysis of the literature, the following is a clear definition of the concept of ‘intertemporal decision‐making in health behaviours’. Intertemporal decision‐making in health behaviours refers to the process by which individuals make trade‐offs and choices between short‐term and long‐term outcomes of health‐related behaviours, with the outcomes encompassing both different aspects (health aspect and nonhealth aspect) and different signs (costs and benefits). Figure 3 shows a conceptual model including the theoretical relationships between antecedents, attributes and consequences. Included articles and defining attributes can be found in Supporting Information 2.

## 5. Discussion

### 5.1. State of the Science

Intertemporal decision‐making in health behaviours is a relatively new concept, with rapidly emerging evidence. To date, most studies in this area follow the theoretical models and methodologies from traditional economics, often substituting health discounts with monetary discounts and even crudely trading health with money. Consequently, approximately 1700 articles have been excluded because of lacking specific health domain characteristics. We encourage more original research on health domain characteristics to ensure the authenticity and accurate determination of information. Then, we reviewed studies that align with health domain criteria and found widespread confusion in the use of the concept. Commonly employed terms to describe this concept include health‐related intertemporal choice, health‐related decision, intertemporal decision‐making in the health domain and intertemporal decision for health. At the same time, specific behavioural decisions such as dietary decisions and exercise decisions are also used. Based on the discussion, we believe that intertemporal decision‐making in health behaviours can better elucidate domain‐specific characteristics, temporal considerations and the nuanced trade‐offs involved in health‐related choices.

### 5.2. Presentation of the Concept

The complexity of intertemporal decision‐making in health behaviours lies in the evaluation of the combined value of different aspects (health and nonhealth), time points (short‐term and long‐term) and signs (costs and benefits) [26, 69]. Generally, choices of single‐dimension options are usually simpler; for example, health outcomes are preferred over nonhealth outcomes, immediate outcomes over delayed outcomes and benefits over costs [9]. However, choices’ complexity increases when options differ across multiple dimensions. An immutable law is that people always underestimate the value of future health. Some people discount delayed health outcomes proportionately, who believe that future health outcomes become less valuable due to the delay [61, 66]; some people systematically ignore long‐term health outcomes and focus more on short‐term aspects [53, 60]. In clinical practice, preference drives the direction of choices. This perspective is substantiated by empirical work from Martínez et al., who further advocate for the integration of patient‐specific preferences into both evidence‐based clinical decisions and nursing service delivery [70]. In general terms, health‐related decision preferences influence people in two opposite ways. When actions involve future health gains, present bias effects can lead people to procrastinate (e.g., wait to take action); when actions involve future health losses, the present bias effect causes people to take action before the appropriate time (e.g., act now and enjoy first) [63]. Consequently, clinical decision for individuals with marked present bias should prioritise interventions that offer immediate feedback (e.g., short‐cycle exercise plans). Furthermore, integrating immediate gratification mechanisms (e.g., retail voucher incentives upon exercise goal attainment) into nursing services frameworks may effectively counteract hyperbolic discounting of future health outcomes.

Interestingly, individuals’ preferences are not static, which depends on whether future health is a loss or gain [26], the duration until the outcome manifests [20], the type of health behaviour involved [45], the magnitude of future health benefits [42] and whether the decision pertains to one’s own health or that of others [62]. This reminds us to assess individual decision preferences in context and to understand the occurrence and change of health behaviours with a flexible perspective. In practice, healthcare providers should develop personalised intervention strategies based on the dynamic changes in patients’ preferences. These strategies should take into account various factors—the nature, magnitude, delay time of health outcomes and the decision‐making entity—to achieve precise care.

Various antecedents and consequences have been identified that contribute to healthcare professionals’ understanding of intertemporal decision‐making in health behaviours. Since decision requires a value trade‐off of health outcomes, health‐related behaviours that produce healthy outcomes must be a prerequisite. Some behaviours with high prevalence rates, such as smoking, exercising, dieting and sexual activity, are currently the focus of research [7, 9, 27]. Meanwhile, the disproportionate outcomes of health behaviours are the substance of intertemporal decision. Existing research demonstrates a high consensus on the long‐term outcomes of health behaviours, while exhibiting subtle discrepancies in short‐term effects. Studies characterise the immediate outcomes like money, time and energy [10]. However, some health‐related outcomes also appear in the short term. For example, antiretroviral treatment in HIV patients also leads to drug side effects in the short term [71]. A simplistic trade‐off between immediate nonhealth outcomes and future health outcomes may oversimplify the complexity of decision processes in health contexts. We advocate for intensified investigation into the immediate outcomes of health behaviours to advance a more comprehensive understanding of the multidimensional nature underlying intertemporal health decisions. When it comes to consequences, the choices made by individuals directly influence their adoption and execution of various health‐related behaviours, specifically, whether these behaviours occur (e.g., smoke or not, unprotected sex or not, etc.) and in what way they occur (e.g., daily smoking quantity, frequency of unprotected sex, etc.) [14, 45]. Meanwhile, many articles involve health outcomes of decision such as physical health, mental health and social adjustment aspects [58, 60]. Therefore, this conceptual model identifies comprehensive health outcomes rather than merely physical health.

The model cases in this study contain all the evidence reviewed and provide a useful reference for concrete examples of how this concept can be implemented in the health sector. For borderline cases, such as wearing a seat belt, its behaviour is more related to the probability of a health outcome and does not mean a trade‐off of specific costs and benefits, and thus they do not fall under intertemporal decision‐making in health behaviours [10]. Borderline cases are introduced primarily to distinguish health intertemporal decision from health risk decisions. Although those two concepts may not be completely unrelated, intertemporal decision‐making focuses on the evaluation and selection of clear values rather than the evaluation and selection of the likelihood of a health outcome. Contrary cases entirely circumvent the process of value trade‐off, in which people’s behaviour is driven by the outside, rather than their own weighing and choice of the health value brought by the action.

## 6. Implications for Nursing Management

The clarification of this concept holds significant potential benefits for nursing management practice. First, this conceptual model assists nursing managers in identifying auditing indicators for preference assessment—specifically health‐related decision preferences. Moreover, several audit‐based measures that promote nursing management practices can serve as references. (a) Guiding preference‐driven personalised care: For example, when interacting with present‐biased patients, prioritising efficient communication helps reduce impatience; when conducting health education for patients with loss aversion, emphasising future health gains proves less impactful than emphasising health losses. (b) Formulating preference‐driven medical resource allocation models: Specifically, patients demonstrating a preference for immediate gratification might benefit from streamlined services like neighbourhood clinics, telehealth consultations and intensive follow‐ups, whereas those prioritising future health outcomes could be guided towards autonomous care models featuring smart health assistants, community prevention initiatives and physician‐supervised self‐management programs. (c) Implementing short‐term bias intervention programs: Managers should identify patients demonstrating ‘short‐sighted’ or ‘present‐biased’. Utilising strategies such as cognitive intervention, anticipatory imagination training and situational future‐thinking can correct their short‐term preferences, thereby facilitating decisions that are beneficial to future health.

The proposal of this concept also provides guidance for the direction of subsequent research. First, a theoretical framework must be developed to address intertemporal decision‐making in health behaviours. Building upon this proposed conceptual model, subsequent studies should empirically test critical hypotheses regarding the relationship between individual characteristics and health‐related decision preferences; the direction by which these preferences drive health behaviours pattern and the intrinsic instability of such preferences. Consequently, research priorities should focus on (a) identifying determinants of health‐related decision preferences, (b) exploring causal relationships between these preferences and behavioural pattern, (c) discovering the sequence of changes in health‐related decision preferences and (d) conducting comparative analyses between gain‐framed versus loss‐framed preferences, as well as variations across distinct health behaviours. Second, developing validated measurement approaches for health‐related decision preferences is crucial to accurately quantify these preferences and discern behavioural‐specific variations. Future research should concurrently pursue (1) creating domain‐general instruments capable of quantifying core preference across various health contexts and (2) designing behaviour‐specific tools for particular health decisions, ensuring precision in capturing behavioural nuance. Finally, future research should explore interventions targeting health‐related decision preferences to improve individuals’ health‐related behaviours. Implementation research should strategically target (1) chronic disease patients’ health management behaviours (e.g., medication adherence, blood glucose monitoring and nutritious diets) demanding sustained long‐term effort and (2) health‐risk behaviours in the general population (e.g., smoking, physical inactivity and poor dietary habits) due to their high prevalence.

## 7. Limitation

There are a few limitations to this concept analysis. First, the articles were restricted to English only, which limits the scope of the investigation. In addition, involving articles that substitute monetary discounts for health discounts may compensate more evidence for loss framework, but given that the focus of this study is intertemporal decision‐making in the health field, we therefore focused on publications that fit the health domain characteristics.

## 8. Conclusion

The application of intertemporal decision‐making in the health field is increasingly prevalent, and it has made outstanding contributions to understanding and predicting patients’ health behaviours. Central to this work is the definition of intertemporal decision‐making in health behaviours. As far as we know, this is a groundbreaking conceptual analysis of ‘intertemporal decision‐making in health behaviours’. Through a systematic search of literature and an analysis guided by Walker and Avant’s method, we identified the antecedents, attributes, consequences and empirical referents of this concept, providing illustrative examples for identification and differentiation. Based on these findings, we also make recommendations for medical practice and future research. We also provide a current, evidence‐based understanding of how individuals make decisions regarding health behaviours, intending for this knowledge to be implemented in future research, education, practice and policy. Judging from the current evidence, the introduction and clarification of this concept may contribute to improving healthcare practice and promoting universal health.

## Funding

This research was supported by the National Natural Science Foundation of China (Grant No. 82273746) and the Graduate Research Innovation Project of Hunan Education Department (Grant No. CX20240324).

## Disclosure

The funding did not influence the study’s design, data collection, analysis, interpretation or writing of the manuscript.

## Ethics Statement

Ethical approval is not required as this is not empirical research.

## Conflicts of Interest

The authors declare no conflicts of interest.

## Supporting Information

Additional supporting information can be found online in the Supporting Information section.

## Supporting information


**Supporting Information 1** Search strategies for databases.

**Supporting Information 2** Included articles and their attributes.

## Data Availability

Data sharing is not applicable to this article as no new data were created or analysed in this study.
