# Supplementary material for: Intertemporal Decision‐Making in Health Behaviours: A Conceptual Analysis
Source: J Nurs Manag. 2026 May 19;2026:9712444. doi: 10.1155/jonm/9712444 (PMC13185753; doi:10.1155/jonm/9712444)
Supplement: Supplementary file 1 — Supporting Information 1 Search strategies for databases. [file JONM-2026-9712444-s002.docx]

Supplementary material 1. Search Strategies for databases

| Database | Project | Keywords | Results |
| --- | --- | --- | --- |
| PubMed | S1 | delay discounting[Title/Abstract] OR intertemporal decision*[Title/Abstract] OR inter temporal decision*[Title/Abstract] OR intertemporal choice*[Title/Abstract] OR inter temporal choice*[Title/Abstract] OR intertemporal decision-making[Title/Abstract] OR intertemporal preference*[Title/Abstract] OR inter temporal preference*[Title/Abstract] OR temporal discounting[Title/Abstract] OR time preference*[Title/Abstract] OR time perspective[Title/Abstract] OR time orientation[Title/Abstract] | 4808 |
|  | S2 | delay discounting[MeSH Terms] | 1415 |
|  | S3 | S1 OR S2 | 5095 |
|  | S4 | health behavio*[Title/Abstract] OR health-related behavio*[Title/Abstract] OR health related behavio*[Title/Abstract] OR health risk behavio*[Title/Abstract] OR risky health behavio*[Title/Abstract] OR alcohol abstinence[Title/Abstract] OR drinking behavior[Title/Abstract] OR sleep hygiene[Title/Abstract]OR smoking[Title/Abstract] OR eating behavior[Title/Abstract] OR sexual behavior[Title/Abstract] OR substance abstinence[Title/Abstract] OR substance abuse[Title/Abstract] OR patient compliance[Title/Abstract] OR treatment refusal[Title/Abstract]OR exercise[Title/Abstract] OR self-examination[Title/Abstract] OR weight control[Title/Abstract] | 720699 |
|  | S5 | health behavior[MeSH Terms] | 370016 |
|  | S6 | S4 OR S5 | 1022405 |
|  | S7 | S3 AND S6 | 775 |
| Embase | S1 | delay discounting':ab,ti OR 'intertemporal decision*':ab,ti OR 'inter-temporal decision*':ab,ti OR 'intertemporal choice':ab,ti OR 'inter-temporal choice*':ab,ti OR 'intertemporal preference*':ab,ti OR 'inter-temporal preference*':ab,ti OR 'time preference':ab,ti OR 'time perception':ab,ti OR 'intertemporal decision-making':ab,ti | 5049 |
|  | S2 | health behavior':ab,ti OR 'health behaviors':ab,ti OR 'high risk behavior':ab,ti OR 'health protect behavior':ab,ti | 94487 |
|  | S3 | smoking:ab,ti OR 'drinking behavior':ab,ti OR eating:ab,ti OR diet:ab,ti OR exercise:ab,ti OR 'body weight loss':ab,ti OR 'sexual behavior':ab,ti OR 'substance use':ab,ti OR 'self care':ab,ti OR vaccination:ab,ti OR 'alcohol consumption':ab,ti OR 'smoking cessation':ab,ti OR cocaine:ab,ti | 1899645 |
|  | S4 | S2 OR S3 | 1916664 |
|  | S5 | S1 AND S4 | 1029 |
| Scopus | S1 | TITLE ( delay AND discounting ) OR TITLE ( time AND discounting ) OR TITLE ( temporal AND discounting ) OR TITLE ( intertemporal AND decision* ) OR TITLE ( intertempotal AND choice* ) OR TITLE ( time AND preference ) OR TITLE ( intertemporal AND preference* ) OR TITLE ( time AND perspective ) OR TITLE ( time AND orientation ) OR TITLE ( intertemporal AND decision-making ) | 10096 |
|  | S2 | KEY ( health AND behavio* ) OR KEY ( health-related AND behavio* ) OR KEY ( health AND risk AND behavio* ) OR KEY ( risky AND health AND behavio* ) OR KEY ( health AND protect* AND behavio* ) OR KEY ( health AND prevention AND behavior* ) | 486107 |
|  | S3 | KEY ( health AND behavio* ) OR KEY ( health-related AND behavio* ) OR KEY ( health AND risk AND behavio* ) OR KEY ( risky AND health AND behavio* ) OR KEY ( health AND protect* AND behavio* ) OR KEY ( health AND prevention AND behavior* ) | 2991368 |
|  | S4 | S2 OR S3 | 3289092 |
|  | S5 | S1 AND S4 | 894 |
| MEDLINE | S1 | MHX=(delay discounting) | 1413 |
|  | S2 | TI=(delay discounting) OR TI=(time discounting) OR TI=(intertemporal decision*) OR TI=(inter-temporal decision*) AND TI=(intertemporal choice*) OR TI=(inter-temporal choice*) OR TI=(intertemporal decision-making) OR TI=(time preference*) OR TI=(temporal preference*) OR TI=(time perception) OR TI=(time perspective ) OR TI=(time orientation) | 5891 |
|  | S3 | S1 OR S2 | 6695 |
|  | S4 | MHX=(health behavior) | 194143 |
|  | S5 | TI=(health behavior*) OR TI=(health-related behavior*) OR TI=(health risk behavior*) OR TI=(health protect behavior*) AND TI=(health prevention behavior*) | 18768 |
|  | S6 | TI=(smoking) OR TI=(drinking) OR TI=(alcohol *use) OR TI=(eating) AND TI=(diet*) OR TI=(esercise) OR TI=(physical activity ) OR TI=(sexual behavior*) OR TI=(weight loss) OR TI=(treat*) OR TI=(self-management) OR TI=(self-care) OR TI=(substance use) OR TI=(vaccination) | 2014134 |
|  | S7 | S4 OR S5 OR S6 | 2167800 |
|  | S8 | S3 AND S8 | 627 |
| CINAHL | S1 | MH delay discounting | 422 |
|  | S2 | AB delay discounting OR AB time discounting OR AB temporal discounting OR AB intertemporal decision* OR AB inter-temporal decision* OR AB intertempotal choice* OR AB inter-temporal choice* OR AB time preference OR AB intertemporal preference* OR AB inter-temporal preference* OR AB time perspective OR AB time orientation | 4041 |
|  | S3 | S1 OR S2 | 4145 |
|  | S4 | MH "health behavior" | 61556 |
|  | S5 | AB health behavio* OR AB Health-Related Behavio* OR AB health Risk Behavio* OR AB risky health behavio* OR AB protect* health behavio* OR AB Health prevention behavior* | 46612 |
|  | S6 | AB alcohol use OR AB ( alcohol abuse or alcoholism or alcohol addiction or alcohol dependence ) OR AB drinking OR AB ( smoking or tobacco or cigarette or nicotine ) OR AB eating OR AB diet* OR AB exercis* OR AB weight loss OR AB sexual behavior OR AB substance use OR AB ( self-management or self-care or self-regulation or self-monitoring ) OR AB vaccination | 485684 |
|  | S7 | S4 OR S5 OR S6 | 548447 |
|  | S8 | S3 AND S7 | 938 |
| APA PsyINFO | S1 | MA delay discounting | 498 |
|  | S2 | DE delay discounting OR DE time discounting OR DE temporal discounting OR DE intertemporal decision* OR DE inter-temporal decision* OR DE intertemporal choice* OR DE inter-temporal choice* OR DE intertemporal preference* OR DE inter-temporal preference* OR DE time preference* OR DE time perspective OR DE time orientation | 2060 |
|  | S3 | S1 OR S2 | 2060 |
|  | S4 | MA health behavior | 17230 |
|  | S5 | DE health behavio* OR DE health-related behavio* OR DE health risk behavio* OR DE risky health behavio* OR DE protect* health behavio* | 446881 |
|  | S6 | DE alcohol use OR DE alcohol abuse OR DE drinking OR DE smoking OR DE eating OR DE diet* OR DE exercis* OR DE weight loss OR DE sexual behavior OR DE substance use OR DE self-management OR DE vaccination | 175541 |
|  | S7 | S4 OR S5 OR S6 | 209712 |
|  | S8 | S3 AND S7 | 264 |
| Econlit with Full Text | S1 | AB delay discounting OR AB time discounting OR AB temporal discounting OR AB intertemporal decision* OR AB inter-temporal decision* OR AB intertemporal choice* OR AB inter-temporal choice* OR AB intertemporal preference* OR AB inter-temporal preference* OR AB time preference* OR AB time perspective OR AB time orientation | 4914 |
|  | S2 | AB health behavio* OR AB health-related behavio* OR AB health risk behavio* OR AB risky health behavio* OR AB health protect* behavio* OR AB protect* health behavio* | 2013 |
|  | S3 | AB alcohol use OR AB alcohol abuse OR AB drinking OR AB smoking OR AB eating OR AB diet* OR AB exercis* OR AB weight loss OR AB sexual behavio* OR AB substance use OR AB self-management OR AB vaccination | 22251 |
|  | S4 | S2 OR S3 | 23722 |
|  | S5 | S1 AND S4 | 230 |
