# Supplementary material for: Intertemporal Decision‐Making in Health Behaviours: A Conceptual Analysis
Source: J Nurs Manag. 2026 May 19;2026:9712444. doi: 10.1155/jonm/9712444 (PMC13185753; doi:10.1155/jonm/9712444)
Supplement: Supplementary file 2 — Supporting Information 2 Included articles and their attributes. [file JONM-2026-9712444-s001.docx]

Supplementary material 2. Included articles and attributes

|  | Authors, Date, Country | Literature type | Antecedents 1.  Health-related behavior | Antecedents 2.  Disproportionate behavioral outcomes | Antecedents 3.  Personal characteristics | Attributes 1. Trade-off between short-term outcomesand long-term outcomes | Attributes 2. Trade-off between other outcomes and health outcomes | Attributes 3. Trade-off between costs and benefits | Attributes4.  Health-related decision preferences drive the direction of choices  ① Delay discounting  ② Time perspective  ③ Ttime orientation/ preference | Attributes5.  Health-related decision preferences are inconstant  ① Sign effec ② Sequence effect  ③ Magnitude effect  ④ Domain efect  ⑤ Object of decision | Consequences 1.  Explicit behavior pattern | Consequence 2.  Health-related outcomes |
| --- | --- | --- | --- | --- | --- | --- | --- | --- | --- | --- | --- | --- |
| 1 | Finkenstaedt et al. , 2024, Germany | Cross-sectional study | Sexual behavior | Immediate reward  and delayed costs | Borderline symptom；  partner desirability;  the perceived sexually transmitted infections risk | "Immediate" VS "Delayed" | "Sexual intercourse" VS " Sexually transmitted infections and unwanted pregancy" | "Reward" VS "Costs" | ① Delay discounting: -People with higher sexual discounts were more likely to engage in compulsive Sexual behavior | N/A | -Immediate sex without a condom or dental dam  -Delayed sex with a condom or dental dam | Sexual healthy |
| 2 | Begho, et al. , 2024, China | Cross-sectional study | Eating  behaviour | Immediate pleasure and long-term health benefits | Convenience; Financial constraints;  nutritional knowledge; social circles; | "Immediate" VS "Long-term" | "Pleasure" VS " Better health" | N/A | ② Time perspective: -Individuals who prioritise the future benefits were less likely to perceive negative health effects from their dietary habits | N/A | -Unhealthy eating  -Healthy eating | Health |
| 3 | Vincent et al. , 2023, UK | Cross-sectional study | Sexual behavior | Immediate reward  and delayed costs | Gonadal hormone levels;  Risks of Pregnancy | "Immediate" VS "Delayed" | "Sexual intercourse" VS "Sexually transmitted infections and unwanted pregancy" | "Reward" VS "Costs" | ① Delay discounting: -People with higher sexual discounts were more likely to engage in risky sexual behavior | ④ Domain efect: food discounting higher than sexual discounting | -Having intercourse later in the cycle  -Having intercourse during menstruation | Women"s health |
| 4 | Mitzel et al. , 2023, USA | Cross-sectional study | Sexual behavior | Immediate reward  and delayed costs | Gender | "Immediate" VS "Delayed" | "Increased pleasure" VS " Sexually transmitted infections" | "Reward" VS "Costs" | ① Delay discounting: -People with higher sexual discounts were more likely to engage in condomless sex | N/A | -Immediate sex without a condom  -Delayed sex with a condom | Sexual healthy |
| 5 | Mistretta et al. , 2023, USA | Cross-sectional study | Useing opioid painkillers | Immediate  pain relief and  long-term aversive side effects | Pain severity; pain cognitions | "Short-term" VS "Long-term" | "Pain relief" VS "Constipation and social withdrawal" | "Reward" VS "Costs" | ① Delay discounting: -People with higher opioid discounts were more likely to engage in using opioid | ① Sign effect: adults with and without chronic pain discounted losses more than gains -“Loss aversion” | -Using opioid immediately  -Not using opioid | N/A |
| 6 | Jin et al. , 2023, China | Cross-sectional study | Dietary behavior | Present efforts and future benefits | Self-control capacity and intention | "Present" VS "Future" | "Time spent and forgoing satisfaction" VS " | "Effort" VS "Benefit" | ③ Ttime orientation: -Patients with future-oriented time perspective are more likely to have self-control in changing dietary behavior | N/A | -Unhealthy dietary behavior  -Healthy dietary behavior | Long-term glycemic control |
| 7 | Bellitti et al. , 2023，USA | Cross-sectional study | Food addiction | Immediate reward  and delayed costs | Levels of hunger | "Immediate" VS "Long-term" | "Taste" VS "Negative  physical and mental health  " | "Reward" VS "Costs" | ① Delay discounting: -People with higher food discounts were more likely to engage in food addiction | N/A | -Eating hyper-palatable food immediately  -Waiting for future healthy food intake | Physical and mental health |
| 8 | Taylor et al. ,2022，USA | Cross-sectional study | Alcohol use | N/A | N/A | "Immediate" VS "Delayed" | N/A | N/A | ① Delay discounting: -People with higher alcohol discounts were more likely to engage in alcohol addiction | N/A | -Immediate alcohol  -Delayed alcohol | Substance addiction |
| 9 | Palacios et al. ，2022，Mexico | Cross-sectional study | Sexual behavior | Immediate reward  and delayed costs | Gender; availability of condoms | "Immediate" VS "Delayed" | "Pleasure" VS " Sexually transmitted infections and unwanted pregnancy" | "Reward" VS "Costs" | ① Delay discounting:  -People with higher sexual discounts were more likely to engage in risky sexual behavior | ② Time-inconsistent: the discount of wome increases with the delay time  ④ Domain efect: men’s discount for condoms use was greater than that for frequency of intercourse and number of sexual partners | -Iimmediate sex without a condom  -Delayed sex with a condom | Number of condomless sexual partners and frequency of sexual intercourse |
| 10 | Fazzino et al. ，2022，USA | Cross-sectional study | Eating behavior | Immediate reward  and long term health costs | Hhyper-palatable food intake, hyper-palatable food craving, and BMIs | "Immediate" VS "Future" | "Taste" VS "Unhealthy BMI" | "Reward" VS "Costs" | ① Delay discounting: discount delayed health consequences.  -People with higher food discounts were more likely to engage in eating palatable food | ② Time-inconsistent: there was a steeper discounting of the delayed reward over time  ③ Magnitude effect: individuals displayed significantly greater discounting of the small magnitude reward relative to the large magnitude reward | -Eating hyper-palatable food immediately  -Eating health food later | BMI |
| 11 | Cheong et al. ，2022，USA | Cross-sectional study | Substance use;  sexual behavior | Short-term rewards  and longer term costs | Aavailability of resources Stability of the environment | "Immediate" VS "Long-term" | "Pleasure" VS "Development of substance use disorders; | "Reward" VS "Costs" | ② Time perspective:  -People with present-perspective are more likely to engage in risky sexual behavior | N/A | -Alcohol use; Having risky sex  -Not drinking alcohol; Having protected sex | Reproductive health |
| 12 | Borland et al. ，2022，Australia | Longitudinal study | Smoking cessation | Immediate costs and future benefit | Economic pressure | "Immediate" VS "Future" | "Nicotine satisfaction and/or nicotine withdrawal" VS "Reduced risk to health, cost savings" | "Costs" VS "Benefit" | ③ Ttime orientation:  -People with future-oriented  are more likely to stop smoking | N/A | -Smoking  -Smoking cessation | N/A |
| 13 | Bable et al. ，2022，USA | Cross-sectional study | Sexual behavior | Immediate gratification and future poor health | Whether using dating app | "Immediate" VS "Future" | "Gratifcation" VS "Better health" | "Reward" VS "Costs" | ① Delay discounting:  -People with higher sexual discounts were more likely to engage in risky sexual behavior | N/A | -Immediate low-quality sex  -Delayed high-quality sex | Sexual healthy |
| 14 | DeHart, W. B.，2020，USA | Cross-sectional study | Smoking | Smaller–sooner outcome and the larger–later outcome | Smoking status | "Immediate" VS "Delayed" | "Consuming combustible tobacco" VS "Psychophysical effects" | N/A | ① Delay discounting：  -Smokers show greater health delay discounting than non-smokers | ④ Domain efect: delay discounts differ for alcohol, food, a temporary health gain, and a temporary cure from a disease | -Smoking  -Not smoking | N/A |
| 15 | Cox et al. ，2020,USA | Cross-sectional study | Cocaine use | Smaller–sooner outcome and the larger–later outcome | N/A | "Delayed" | "Negitive health consequence" | N/A | ① Delay discounting:  -Cocaine users show greater delay discounting than non-cocaine users | ①Sign effect：the discount for rewards is greater than the discount for losses;  ③ Magnitude effect：delay discounting systematically decreased as amount increased for health gains;  ④ Domain efect: cocaine-users discounted cocaine-related outcomes most steeply comparing health value | -Using cocaine  -Not using cocaine | Cardiac, pulmonary, and hepatic disease |
| 16 | Pozolotina et al. ，2019, Norway | Cross-sectional study | Physical activity;  smoking; eating | Small present rewards and  larger health impact in the long run | N/A | "In the present time" VS "In the far future" | "Physical effort, time, and financial investment" VS | "Costs" VS "Reward"; "Reward" VS "Costs" | ③ Time orientation:  -Consideration of immediate consequences was linked to unhealthy behaviors; -Consideration of future consequences was associated with healthy behaviors | N/A | -Unhealthy behavior:smoking and unhealthy eating  -Healthy behavior: physical activity and healthy eating | N/A |
| 17 | Madsen et al., 2019, Denmark | Review | Diabetes self-management behaviours | Incurring costs now and health benefits later | N/A | "Immediate" VS "Delayed" | "Money, effort and time" VS "Better health" | "Costs" VS "Benefit" | ① Delay discounting:  -People with higher helth delay discounts were more likely to engage in better self-management | N/A | -More self-management  -Less self-management | HbA1c and diabetes related complications |
| 18 | Baird et al. ，2019，UK | Cross-sectional study | Self-monitoring of blood glucose | Short-term costs and future benefit | N/A | "Short-term" VS "Long-term" | "Inconvenience, discomfort, diffi culty, or fear of a “bad” monitoring result" VS "Lower likelihood of kidney failure, stroke, and heart attack" | "Costs" VS "Benefit" | ② Time perspective:  -A more balanced time perspective was associated with more frequent self-monitoring of blood glucose | N/A | -Monitoring blood glucose regularly  -Monitoring blood glucose irregularly | HbA1c and diabetes related complications |
| 19 | Albelwi et al. ，2019，UK | Experimental study | Exercise | Short-term consequences and long-term consequences | Exercise motives；running training | "Short-term" VS "Long-term" | "Pleasure, eating highly palatable caloric foods, watching television" VS "Physical fitness, appearance, weight" | "Sacrifice" VS "Benefit" | ① Delay discounting: the health values of exercise are time-discounted  -The higher the exercise discount, the lower the activity level | N/A | -Exerciseing  -Not esercising | Risk of death  cardiovascular disease, diabetes and cancer |
| 20 | Tórtora et al. ，2018，UK | Cross-sectional study | Eating behavior | Immediate consequences and future consequences | Attidute to health; interest in nutritional information; education level | "Immediate" VS "Future" | "Pleasure of eting tasty food" VS "Adverse health effects" | "Benefit" VS "Costs" | ③ Time orientation: t  -Coustom with greater consideration of future consequences always have a healther diet | N/A | -Unhealthy diet  -Healthy diet | Depression and Obesity |
| 21 | Karl et al. , 2018，Germany | Cross-sectional study | Self-management: diet, exercise, medication, blood sugar, and foot care | Immediate smaller (dis)utility activities  and utility that bigger but distant in time | Age; education level | "Immediate" VS " Distant in time" | "Self management efforts" VS "Diabetic  complications" | N/A | ③Time preference: high time preference is associated with lower overall self-management | N/A | -Worse self-management  -Better self-management | Disease progression and comorbidity |
| 22 | Jones et al. , 2018，USA | Cross-sectional study | Sexual behavior | Smaller, sooner reward  and long-term  costs | Using of pre-exposure prophylaxis | "Short-term" VS "Long-term" | "Pleasure" VS "Sexually transmitted infections" | "Reward" VS "Costs" | ① Delay discounting:  -People with a higher sexual discount are more likely to condomless anal intercourse | N/A | -Condomless anal intercourse -Condom anal intercourse | HIV risk |
| 23 | Collado et al., 2017, USA | Cross-sectional study | Sexual behavior | N/A | Gender; the desire for sexual partner; | "Immediate" VS "Delayed" | N/A | N/A | ① Delay discounting:  -Pvioreople with higher sexual discounts were more likely to engage in risky sexual behavior | N/A | -Smaller-immediate durations of sex  - Larger-delayed durations of sex | Sexual health |
| 24 | Tompkins et al., 2016, USA | Cross-sectional study | Opioid use | Immediate reward  and future punishment | Motivation of behavior | "Immediate" VS "In the feture" | "Pain relief, feeling high, elevation in mood " VS "Greater pain" | "Reward" VS "Punishment" | ① Delay discounting:  -High discounting of pain relif was positive with opioid misuse  -High discounting of additional pain was negitive with opioid misuse | ① Sign effect: discount of pain relief was bigger than discount of additional pain  -"Loss aversion"  ②Time inconsistency: there was an increase in delayed reward discounting during opioid withdrawal | -Opioid misuse  -Use opioids in moderation | Pain |
| 25 | Koffarnus et al., 2016, USA | Cross-sectional study | HIV risk behavior | Immediate reward  and delay reward | Levels of cocaine use; access to condoms; intelligence | "Immediate" VS "Delayed" | "Pleasure" VS "Improved health, interpersonal relationships, employment opportunities" | N/A | ① Delay discounting: t  -Pvioreople with higher sexual discounts were more likely to engage in risky sexual behavior  HIV risk behavior | N/A | -Risky sexual behaviors related to drug use  -Safe sexual behavior | Sexual health |
| 26 | Friedel et al., 2016, USA | Cross-sectional study | Smoking | N/A | Drinking; smorking; level of formal education | N/A | N/A | N/A | ① Delay discounting: -Smokers demonstrate steeper discounting across a range of delayed outcomes | ① Sign effect: gains discounted more than losses;  ② Time-inconsistent: short delays produce relatively large decreases in value; | -Smoking  -Smoking cessation | Health and death |
| 27 | Barlow et al., 2016, UK | Review | Unhealthy diets | Smaller immediate rewards and larger rewards available after a delay | Socioeconomic factors；cognitive function；anguage development, home learning environments, parenting style and beliefs and health | "Immediate" VS "Long-term" | "Satisfaction" VS "Better health" | N/A | ① Delay discounting:  -There was a higher time discount rates in persons consuming un-healthy diets and overweight and obese individuals  -Higher time discount rates in persons consuming un-healthy diets and overweight and obese individuals | N/A | -Unhealthy diets  -Healthy diets | Overweight and  obesity |
| 28 | Lim et al., 2015, USA | Cross-sectional study | Lose weight | N/A | N/A | "Immediate" VS "Wait" | "Pleasure from delicious but calorically dense treats" VS " Overweight or obese" | N/A | ① Delay discounting:  -A steeper temporal discounting of delayed weight-loss repre- sents decision impulsivity for immediate weight-loss | ③ Magnitude effect: the temporal discounting rates linearly decreased with greater amounts of weight-loss | -Delayed weight-loss  -Immediate weight-loss | The balance of healthy energy |
| 29 | Griva et al., 2015, UK | Cross-sectional study | Smoking, exercise and eating | N/A | Perception of health condition | N/A | N/A | N/A | ② Time perspective:  -A future perspective seems to account for motivating individuals to adopt healthier behaviors including exercising | N/A | -Smoking  -Exercise and eating | Healthy status |
| 30 | Dassen et al., 2015，Netherlands | Cross-sectional study | Eating behavior | Short-term benefits and long-term benefits | N/A | "Short-term" VS "Long-term" | "Taste, satiation" VS "Health and body weight" | N/A | ③ Time orientation:  -A food-specifific measure of consideration of future consequences is related to healthy eating | N/A | -Unhealthy eating  -Healthy eating | Overweight |
| 31 | Story et al., 2014, UK | Review | Unhealthy behavior | A small improvement in health occurring sooner, and a larger improvement at a delay | N/A | "Sonner"  VS "Delay" | "Smaller improvement in health" VS "Larger improvement in health" | N/A | ① Delay discounting:  -High discount rates for food or drug rewards are associated with several unhealthy behaviors | ① Sign effect: there was a positive discounting for health improvement but negative discounting for fleeting illnesses | -Unhealthy behavior  -Healthy behavior | Illness and health imporvement |
| 32 | Mahboub et al., 2014, Iran | Review | Addiction;  smoking;  diet; lifestyle | N/A | N/A | "Today" VS "Future" | N/A | "Gain" VS "Loss" | ① Delay discounting:  -Time preference for own health and time preference for social health | ⑤ Domain efect: time preference for own health and time preference for social health are different | N/A | N/A |
| 33 | Herrmann et al., 2014, USA | Cross-sectional study | Sexual behavior | Immediate reinforce- ment  and delayed punishment | Perceived risk of exually transmitted disease | "Immediate" VS "Delayed" | "Reinforcement" VS "HIV/STI infection, unintended pregnancy" | "Reinforcement" VS "Punisment" | ① Delay discounting:  -Greater discounting of delayed condom-protected sex may partially explain the elevated rates of risky sexual behavior | N/A | -Condomless sex  -Condom-protected sex | HIV transmission among opioid-dependent women |
| 34 | Cavaliere et al., 2014, Italy | Cross-sectional study | Dietary | Present gratification and health improvements in the future | Educated individuals；social classes; age | "Immediate" VS "Delayed" | "Gratifification" VS "Health improvement" | "Reward" VS "Costs" | ③Time preference for food：  -Consumers characterized by high time prefernce are more likely to make unhealthy food choices | N/A | -Unhealthy eating  -Healthy eating | A healthy weight or excess weight |
| 35 | Van et al., 2013, Netherlands | Cross-sectional study | Eating behavior and exercise | Immediate outcomes and future outcomes | N/A | "Immediate" VS "Future" | "Pleasure" VS "Health effects" | N/A | ③Time orientation:  -Consideration of future exercise consequence predicted exercising behavior | N/A | -Self-controlled eating behavior or not  -Planning exercise or not | Obesity or not |
| 36 | Sansone,et al., 2013, Canada | Cross-sectional study | Smkiong | Immediate rewards and future health costs | Awareness of specific health risks | "Immediate" VS "Future" | "stress reduction, improved concentration" VS " Health" | "Reward" VS "Costs" | ③Time orientation:  -People with future-oriented were more likely to be non-smokers | N/A | -Smoking  -Not smoking | Health |
| 37 | Peretti al., 2013, France | Cross-sectional study | Smoking | Immediate benefits and the delayed costs | Socioeconomic status; education level | "Immediate" VS " Delayed" | N/A | "Reward" VS "Costs" | ③Time preference:  -Present-oriented people are more prone to cigarette smoking | N/A | -Smoking  -Quitting | smoking-related cancer |
| 38 | Brown et al., 2013, Australia | Longitudinal study | Smoking cessation | Present-day benefits  and long-term benifits | Whether quit Smoking | "Present-day" VS "Long-term" | "Relaxation and coping" VS "Decreasing the risk of disability and disease" | N/A | ③Time preference:  -Time preference temporally predicts successful smoking cessation | N/A | Successful cessation or not | N/A |
| 39 | Rosin et al.，2012, Israel | Review | Weight‐loss; dieting | Immediate costs and delayed rewards; immediate rewards but adelayed costs | N/A | "Immediate" VS " Delayed" | "Gratifcation" VS  "Gaining weight" | "Reward" VS "Costs"  "Costs" VS "Reward" | ③Time preference: bias effect causes people to procrastinate or to act before the proper time | ⑤ Domain efect: time preference for own health and time preference for others are different | N/A | N/A |
| 40 | Johnson et al.，2012，USA | Cross-sectional study | HIV risk behavior | Immediate and brief effects and delayed improve- ments in health | gender; the desire for sexual partner; perception of partners’sexually transmitted disease status | "Immediate" VS "Delayed" | "Increased risk of HIV and other health problems" VS "A healthier life" | "Costs" VS "Benifits" | ① Delay discounting:  -Discounting of delayed sexual rewards is a critical variable strongly affecting HIV sexual risk behavior | N/A | Unprotected sex or delayed protected sex | Prevent or acquire AIDS |
| 41 | Hall et al., 2012, Canada | Longitudinal study | Weight management behaviors: dietary behavior and physical activity | N/A | N/A | " Present" VS "Extended period of time" | N/A | N/A | ③ Time orientation:  -Future-oriented predicts change in self-management behaviors over the early stages following diagnosis of T2DM | N/A | Decreased fatty food consumption and increased levels of physical activity | The course of the disease |
| 42 | Johnson et al., 2007, USA | Longitudinal study | Smoking | Enjoy smoking and health years from now | Severity of smoking | "Now" VS "Years from now" | "Enjoy smoking" VS "Improved health" | "Gains" VS "Losses" | ① Delayed discounting:  -Delay discounting of cigarettes is assciated with smking rate in heavy smokers | ③ Magnitude effect: smaller magnitudes discounted more than larger magnitudes was found for cigarettes;  ④ Domain efect: light smokers discounted cigarettes significantly more than health | Smoking or not | N/A |
| 43 | James et al., 2006, USA | Cross-sectional study | Alcohol use; drug use; tobacco use; sex behaviors; exercise | Immediate or long-term health consequences | N/A | "Immediate" VS "Long-term" | "Meet desires" VS "Health consequences" | N/A | ② Time perspective:  -Young adults with a future time perspective tended to engage in fewer risky health behaviors and were more likely to adopt health protective behaviors. | N/A | -Health risky behavior  -Health protective behaviors | Health condition |
| 44 | Gurmankin et al., 2006, USA | Case-control study | BRCA1/2 testing; annual mammography | Immediate benefits and delayed costs | Levels of education; incomes | "Immediate" VS "Delayed" | "Pleasure or convenience" VS "Improvements in health or survival" | "Benifits" VS "Costs" | ③ Time orientation:  -Future time preference was associated with BRCA1/2 testing and adherence to annual mammography | ⑤ Domain efect: time preference for own health and time preference for others are different | N/A | Improvements in health or survival |
| 45 | Wiggers et al., 2005, Netherlands | Randomized clinical trial | Smoking cessation | N/A | N/A | "Now" VS "Years from now" | "Duration of survival" VS "Expectations of improved health, increased life expectancy and quality of life" | "Costs" VS "Benifits" | ① Delayed discounting:  -Health preferences strongly predict smoking cessation on both point prevalence variables, namely 24-h quit and 7-day quit | N/A | Smoking or quitting | Health and the quality of live |
| 46 | Odum, A. L.，2002，USA | Cross-sectional study | Smoking | N/A | Smoking status | "Immediate" VS "Delayed" | N/A | "Reward" VS "Costs" | ① Delayed discounting:  -Cigarette smokers dis-counted health gains more steeply than never-smokers | ① Sign effect: smokers and ex-smokers discounted health losses more steeply than health gains | Smoking or quitting | N/A |
| 47 | Chapman et al., 2001, USA | Cross-sectional study | Health preventive behaviors | Short-term cost and long-term gain | N/A | "Immediate" VS "Delayed" | "Pain of the injection" VS "Reduce the risk of flu"; | "Costs" VS "Benifits" | ① Delayed discounting:  -Health time preferences were not correlated with flu shot acceptance | N/A | Getting an influenza vaccination, taking medication to control hypertension, | N/A |
| 48 | Chapman et al., 1999, USA | Cross-sectional study | Getting an influenza vaccination | An up-front cost and a delayed benefit | N/A | "Immediate" VS "Delayed" | "Cost, inconvenience, pain of the injection" VS "Prevent influenza" | "Costs" VS "Benifits" | ① Delayed discounting:  -People with future-oriented time preferences would be more likely to accept a free influenza vaccination | N/A | Accepting or rejecting the flu vaccine | The risk of infect influenza |
| 49 | Bickel et al., 1999, USA | Cross-sectional study | Somking | Immediate drug intoxication or transient withdrawal symptoms and a variety of delayed rewards | Smoking status | "Immediate" VS "Delayed" | N/A | "Reward" VS "Costs" | ① Delayed discounting:  -Chronic cigarette smoking could produce steeper discounting | N/A | N/A | Drug-dependent |
